# Central Role of Mitochondrial Oxidative Stress in the Pathophysiology of Disorders

**DOI:** 10.3390/biomedicines13122881

**Published:** 2025-11-26

**Authors:** Guilhian Leipnitz, André Quincozes-Santos

**Affiliations:** 1 Post-Graduation Program in Biological Sciences: Biochemistry, Department of Biochemistry, Universidade Federal do Rio Grande do Sul, Porto Alegre 90035-003, RS, Brazil; guilhian@ufrgs.br (G.L.); andrequincozes@ufrgs.br (A.Q.-S.); 2 Post-Graduation Program in Neurosciences, Universidade Federal do Rio Grande do Sul, Porto Alegre 90035-003, RS, Brazil; 3 Post-Graduation Program in Biological Sciences: Physiology, Universidade Federal do Rio Grande do Sul, Porto Alegre 90035-003, RS, Brazil

Mitochondria are dynamic organelles that play an essential role in cellular redox homeostasis and bioenergetics. Their integrity and function depend on quality control processes, including biogenesis, fusion, fission, and mitophagy. Therefore, the orchestrated functioning of these processes ensures adequate ATP production and the generation of low levels of reactive oxygen species (ROS). However, changes in mitochondrial function are commonly related to the pathophysiology of several diseases.

Mitochondrial biogenesis is the process that synthesizes mitochondria through the growth and division of existing ones, increasing their overall number and mass [1]. This process is primarily regulated by peroxisome proliferator-activated receptor gamma coactivator 1-alpha (PGC-1α) [1]. PGC-1α activation stimulates different nuclear transcription factors, such as nuclear respiratory factor-1 (NRF-1), nuclear respiratory factor-2 (NRF-2) and estrogen-related receptor-α (ERR-α), as well as the expression of TFAM, the final effector of mtDNA transcription and replication [1]. Mitochondrial fusion is mainly controlled by mitofusins 1 and 2 (Mfn1 and Mfn2) and OPA1 (for fusion), whereas fission is controlled by Drp1 and Fis1 (for fission). Mitochondrial fusion merges two mitochondria into a single organelle, whereas fission divides a single mitochondrion into two smaller ones, often aiming to eliminate a damaged organelle. These processes help maintain an adequate number of functional mitochondria. Interestingly, ROS have been described as important regulators of mitochondrial biogenesis and dynamics by modulating the expression and activity of the proteins involved in these processes [2,3].

The relationship between ROS levels, bioenergetics and mitochondrial biogenesis in cardiomyocytes is the main theme of the study by Spinelli et al. (2024) [4]. This article demonstrates that the abscisic acid (ABA)/LANCL1-2 hormone/receptor system may regulate ROS levels in cardiomyocytes, enhancing their capacity to cope with oxidative stress, and that this modulation is dependent on estrogen-related receptors (ERRs). A previous report from the same research group showed that the LANCL/AMPK/PGC-1α/ERRα axis stimulates mitochondrial biogenesis and improves energy production [4], suggesting that this signaling pathway is responsible for controlling ROS levels.

Disturbances in mitochondrial quality control processes associated with oxidative stress and bioenergetic dysfunction have been shown to underlie the pathophysiology of different genetic disorders. Silveira et al. (2024) [5] demonstrated that intracerebral injection of 3-hydroxy-3-methylglutaric acid (HMG), the major metabolite accumulated in 3-hydroxy-3-methylglutaryl-CoA lyase deficiency, disrupts antioxidant defenses, the citric acid cycle, and electron transport chain function and induces mitochondrial fission in the brain of rats. These deleterious effects elicited by HMG also impaired the neurodevelopment of animals. In this context, increasing evidence has revealed that impairment of the electron transport chain (ETC) leads to the generation of mitochondrial ROS, which may induce an increase in intracellular calcium levels and consequently open the mitochondrial permeability transition pore [6,7,8]. These mechanisms have been shown to induce mitochondrial fission in various pathologies [6].

Mitochondrial dysfunction has also been implicated in metabolic diseases. A study revealed that a cocktail containing 17β-estradiol, leptin, IL-6 and TNFα, which is known to simulate the obesity-related inflammation condition in postmenopausal women, increased the aggressiveness of 3D cultures of breast cancer cell lines by reducing mitochondrial and antioxidant-related markers [9]. Notably, this treatment also reduced the sensitivity of 3D-derived T47D cells to tamoxifen and paclitaxel treatment [9].

Non-alcoholic fatty liver disease (NAFLD) and non-alcoholic steatohepatitis (NASH) are metabolic diseases associated with inflammation and mitochondrial alterations. Human and animal studies have shown that alterations in the gut microbial community composition frequently occur in individuals with NAFLD [10,11], which may contribute to the inflammation and mitochondrial dysfunction observed in this condition. Metabolites produced by the gut microbiota may interact with mitochondrial function, genes, and inflammatory factors. Disruption of the gut microbiota also results in elevated levels of lipopolysaccharide (LPS) in the blood or liver, triggering hepatic inflammation [12]. Importantly, the gut microbiota and their metabolites have also been demonstrated to play crucial roles in processes such as mitochondrial biogenesis, metabolism, and modulation of oxidative stress [13,14]. Bahitham et al. (2024) [15] provide a comprehensive review of this topic in this Special Issue.

Another review in this Special Issue discusses the association between mitochondrial dysfunction and the development of atrial fibrillation (AF) (Mauriello et al., 2024) [16]. AF is a cardiac arrhythmia characterized by anatomical and functional alterations in cardiomyocytes caused by alterations in ionic fluxes and cardiomyocyte electrophysiology. Evidence for the involvement of mitochondrial perturbations in the pathophysiology of AF comes from reports showing that drugs and nutraceuticals that indirectly improve mitochondrial homeostasis may represent a therapeutic approach for the treatment of cardiac arrhythmias.

This Special Issue provides a relevant collection of articles that highlights the role of mitochondrial dyshomeostasis, with emphasis on ROS production and oxidative stress, in the pathophysiology of metabolic disorders. This Special Issue also focuses on the influence of ROS in the modulation of other mitochondrial functions, such as mitochondrial quality control and inflammatory response. We believe that these articles may stimulate research on the central role of mitochondria in the pathological mechanisms of disorders and the development of adjuvant therapeutic strategies for these conditions, particularly metabolic diseases.

## Abbreviations

The following abbreviations are used in this editorial:

HMG	3-hydroxy-3-methylglutaric acid
AF	Atrial fibrillation
ERR-α	Estrogen-related receptor-α
ERRs	Estrogen-related receptors
ETC	Electron transport chain
LPS	Lipopolysaccharide
MFN1	Mitofusin 1
MFN2	Mitofusin 2
NAFLD	Non-alcoholic fatty liver disease
NRF1	Nuclear respiratory factor-1
NRF2	Nuclear respiratory factor-2
PGC-1α	Peroxisome proliferator-activated receptor gamma coactivator 1-alpha
ROS	Reactive oxygen species
